# Xerostomia, the perception of general and oral health and health risk behaviours in people over 65 years of age

**DOI:** 10.1186/s12877-022-03667-3

**Published:** 2022-12-19

**Authors:** Alba Pérez-Jardón, Mario Pérez-Sayáns, Manuel Peñamaría-Mallón, Eva Otero-Rey, Eugenio Velasco-Ortega, José López-López, José María Martínez-González, Andrés Blanco-Carrión

**Affiliations:** 1 Oral Medicine, Oral Surgery and Implantology Unit (MedOralRes). Faculty of Medicine and Dentistry, Universidade de Santiago de Compostela, 15705 A Coruña, Spain; 2 ORALRES group. Instituto de Investigación Sanitaria de Santiago (IDIS), 15706 Santiago de Compostela, Spain; 3 Department of Stomatology, Faculty of Dentistry, University of Seville, C/Avicena s/n, 41009 Seville, Spain; 4 Oral Health and Masticatory System Group-IDIBELL, Faculty of Medicine and Health Sciences, Odontological Hospital University of Barcelona, University of Barcelona, 08907 Barcelona, Spain; 5 Department of dental Clinical Specialties, Faculty of dentistry, Complutense University of Madrid, 28040 Madrid, Spain

**Keywords:** Xerostomia, Health, Lifestyle, Oral health, Quality of life

## Abstract

**Background:**

This study investigated the association between xerostomia and health risk behaviours, general and oral health and quality of life.

**Methods:**

A cross-sectional study involving 800 adults over 65 years of age residing in Spain using a computer-assisted telephone questionnaire. The severity of xerostomia was assessed through the Xerostomia Inventory (XI). Both univariate and adjusted multinomial logistic regression were used to determine the risk (OR) of xerostomia.

**Results:**

The sample comprised of 492 females (61.5%) and 308 males, with a mean age of 73.7 ± 5.8 years. Some, 30.7% had xerostomia: 25.6% mild, 4.8% moderate and 0.3% severe, the majority being female (34.8% vs 24%; *p* = 0.003). The mean XI was 24.6 ± 6.3 (95% CI 19.2–24.8) for those with poor health, whereas it was 17.4 ± 6.3 (95%CI 16.1–18.6) in those reporting very good health (*p* < 0.001). This difference was also observed in terms of oral health, with the XI mean recorded as 14.7 ± 10.7 for very poor oral health and 6.4 ± 5.4 for those with very good health (*p* = 0.002). Logistic regression showed that the highest OR for xerostomia was observed among adults with poor general health (2.81; 95%CI 1.8–4.3; *p* < 0.001) and for adjusted model the OR was still significant (2.18; 95%CI 1.4–3.4; *p* = 0.001). Those who needed help with household chores had 2.16 higher OR (95%CI 1.4–3.4; *p* = 0.001) and 1.69 (95%CI 1.1–2.7; *p* = 0.03) in the adjusted model. Females had a higher risk of suffering from xerostomia than males.

**Conclusion:**

The strong association between xerostomia and the general and oral health status of older adults justifies the need for early assessment and regular follow-up.

**Supplementary Information:**

The online version contains supplementary material available at 10.1186/s12877-022-03667-3.

## Background

The world’s population is ageing at a much faster rate than ever before, and it is estimated that between 2015 and 2050, the proportion of the world’s population over 60 years of age will increase from 12 to 22% [1]. Xerostomia (or the feeling of dry mouth) mainly affects older people and may be due to a variety of underlying etiologies [2]. Older people have more comorbidities, meaning that a high proportion are polymedicated. It is estimated that so-called “polypharmacy” affects 40–50% of the older population in high-income countries [3], and it is a well-known cause of hyposalivation and xerostomia [2].

Alcohol use disorders in the geriatric population are considered to be the “invisible epidemic” [4]. The European Union has the highest rate of alcohol consumption in the world, with a seemingly low perception of the associated risks [5]. Alcohol ingestion inhibits the release of the antidiuretic hormone, resulting in body dehydration [6], likewise, it also causes salivary gland atrophy and is one of the main causes of sialadenosis [7]. Smoking also has this same effect, and preliminary studies have shown that long-term smoking is significantly associated with hyposalivation [8]. Profound and complex interactions exist between nutrition and oral health [9], however, to our knowledge, no existing studies have considered the relationship between diet in older people and the sensation of dry mouth, although Machowicz et al. [10] associated adherence to a Mediterranean diet with a lower probability of suffering from primary Sjögren’s Syndrome.

The relationship between oral and general health has been widely discussed in scientific literature and it is known that poor oral health can increase the risk of certain physical disorders [11]. A meta-analysis published in 2021 found a positive association between poor general health and poor oral health-related quality of life among older adults [12]. In a systematic map of systematic reviews that examined current knowledge about older persons’ oral health status and dental care, it is concluded that there is an urgent need for research within most domains in geriatric dentistry [13].

The aim of this study was to analyse the association between xerostomia and health risk behaviours, general and oral health and oral health-related quality of life among a large representative sample of adults over 65 years of age.

## Methods

Data came from the 2020 SEGER (Spanish society of Gerodontology) Survey, a national survey following the STROBE guidelines for observational studies [14].

The target population included men and women over the age of 65, with significant representation throughout Spain. Simple random sampling was performed at the national level, with proportional stratification by geographic area. Data were collected anonymously, and the study was granted an exemption from requiring ethics approval by the Bioethics Committee of the University of Santiago de Compostela.

### Survey design

A computer-assisted telephone survey (CATI research method: Computer-Assisted Telephone Interviewing) was conducted using a structured questionnaire (interview length: 16 minutes). This questionnaire comprised five sections that includes information related with: Social-demographic data; General and oral health; Oral problems during 2019; Tobacco and alcohol consumption and Dietary habits.

### Variables

The Xerostomia Inventory (XI) was used to assess dry mouth sensation, designed in 1999 [15] using the validated Spanish version [16]. The ranges of values associated with each degree were as follows: 0–11: No xerostomia, 12–22: Mild xerostomia, 23–33: Moderate xerostomia, and 34–44: Severe xerostomia [17]. In relation to health risk behaviours, tobacco use has been assessed (former or current, number of cigarettes smoked and, for ex-smokers, years since they gave up smoking) as well as alcohol consumption (type of alcoholic beverages and frequency) and dietary habits (frequency of vegetables, legumes, fruit, white meat (chicken, rabbit, turkey), red meat (beef, pork, lamb) and daily drinking water). In relation to general and oral health, concerns about own general and oral health status and perception of own general and oral health were assessed following a 5-point Likert scale. With regard to oral health-related quality of life, it was assessed in terms of oral problems during 2019.

The variables eligible for inclusion in the model, namely “general health” and “oral health”, were split into two by considering “poor or very poor” as poor, and “acceptable, good or very good” as good, for the multilevel binary logistic regression.

### Statistical analysis

Contingency tables analysed the associations between categorical variables using a chi-squared test. Parametric statistics were used to describe the differences in the means, using the ANOVA test with Bonferroni post-hoc correction for comparisons with more than two elements. Both univariate and adjusted multinomial logistic regression were used to determine the Odds Ratio (OR) of xerostomia. A Directed Acyclic Graph (DAG) has been constructed by using the version 0.2.7 of the R package ggdag (R Core Team, 2022). It provides a visual representation of causal relationships among the set of variables involved in the adjusted multinomial logistic regression. The data were analyzed with the SPSS v.28.00 (IBM, Madrid, Spain). The significance level was *p* ≤ 0.05.

## Results

**Fig. 1:**
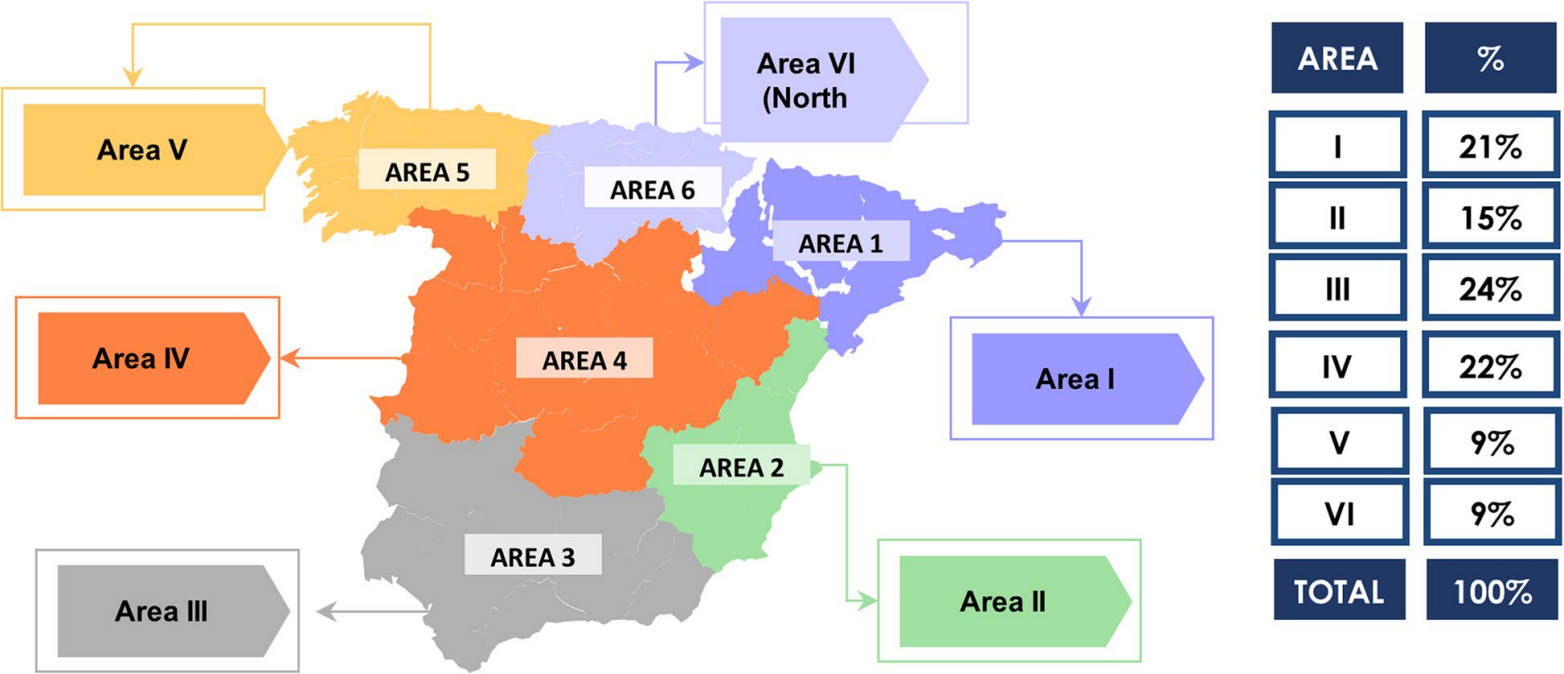
Geographical distribution of participants

The sample comprised 492 females (61.5%) and 308 males (38.5%) with a mean age of 73.7 years (SD = 5.8). The complete descriptive data are in Table 1 and their territorial distribution is outlined in Fig. 1.

**Table 1:** Sample descriptive data

		N	%	Xerostomia Inventory					
				No	Mild	Moderate	Severe	Xeros total	p
**Section I: Social-demographic data**									
Gender	Male	308	38.5	234 (76.0)	67 (21.8)	7 (2.3)	0 (0)	74 (24.0)	0.003
	Female	492	61.5	321 (65.2)	138 (28)	31 (6.3)	2 (0.4)	171 (34.8)	
Age	65 to 74 years	460	57.5	321 (69.8)	115 (25.0)	23 (5.0)	1 (0.2)	139 (30.2)	0.944
	Over 74 years	340	42.5	234 (68.8)	90 (26.5)	15 (4.4)	1 (0.3)	106 (31.2)	
Area	North East	158	19.8	109 (69.0)	41 (25.9)	8 (5.1)	0 (0)	49 (31.0)	0.649
	East	117	14.6	82 (70.1)	30 (25.6)	5 (4.3)	0 (0)	35 (29.9)	
	South	120	15.0	84 (70.0)	28 (23.3)	8 (6.7)	0 (0)	36 (30.0)	
	Centre	177	22.1	122 (68.9)	48 (27.1)	6 (3.4)	1 (0.6)	55 (31.1)	
	Northwest	79	9.9	55 (69.6)	24 (30.4)	0 (0)	0 (0)	24 (30.4)	
	North Central	75	9.4	53 (70.7)	16 (21.3)	6 (8.0)	0 (0)	22 (29.3)	
	Islands and autonomous cities	74	9.3	50 (67.6)	18 (24.3)	5 (6.8)	1 (1.4)	24 (32.4)	
Education	Uneducated	43	5.4	30 (69.8)	12 (27.9)	1 (2.3)	0 (0)	13 (30.2)	< 0.001
	Primary Education	287	35.9	183 (63.8)	89 (31.0)	14 (4.9)	1 (0.3)	104 (36.2)	
	Higher Education	227	28.4	170 (74.9)	46 (20.3)	11 (4.8)	0 (0)	57 (25.1)	
	First Level Vocational Education and Training / Secondary Education 1	80	10.0	51 (63.7)	24 (30.0)	5 (6.3)	0 (0)	29 (36.3)	
	Higher-level Vocational Education and Training Secondary Education 2	150	18.8	114 (76.0)	30 (20.0)	6 (4.0)	0 (0)	36 (24.0)	
	Do not know / No answer given	13	1.6	7 (53.8)	4 (30.8)	1 (7.7)	1 (7.7)	6 (46.2)	
Employment	Employed	623	77.9	439 (70.5)	152 (24.4)	31 (5.0)	1 (0.2)	184 (29.5)	0.065
	Self-employed	129	16.1	88 (68.2)	35 (27.1)	6 (4.7)	0 (0)	41 (31.8)	
	Other	48	6.0	28 (58.3)	18 (37.5)	1 (2.1)	1 (2.1)	20 (41.7)	
Retired	No	84	10.5	57 (67.9)	20 (23.8)	7 (8.3)	0 (0)	27 (32.1)	< 0.001
	Yes	710	88.8	495 (69.7)	183 (25.8)	31 (4.4)	1 (0.1)	215 (30.3)	
	Do not know / No answer given	6	0.8	3 (50.0)	2 (33.3)	0 (0)	1 (16.7)	3 (50.0)	
Do you live alone or with someone else?	I live alone	219	27.4	137 (62.6)	72 (32.9)	9 (4.1)	1 (0.5)	82 (37.4)	< 0.001
	I live with someone else	572	71.5	412 (72.0)	131 (22.9)	29 (5.1)	0 (0)	160 (28.0)	
	Do not know / No answer given	9	1.1	6 (66.7)	2 (22.2)	0 (0)	1 (11.1)	3 (33.3)	
Household chores	I require help with household chores	91	11.4	49 (53.8)	34 (37.4)	8 (8.8)	0 (0)	42 (46.2)	< 0.001
	I am self-sufficient	701	87.6	502 (71.6)	168 (24.0)	30 (4.3)	1 (0.1)	199 (28.4)	
	Do not know / No answer given	8	1.0	4 (50.0)	3 (37.5)	0 (0)	1 (12.5)	4 (50.0)	
**Section II: General and oral health**									
Are you concerned about your general health?	Not at all	144	18.0	124 (86.1)	18 (12.5)	2 (1.4)	0 (0)	20 (13.9)	0.001
	Slightly	128	16.0	85 (66.4)	34 (26.6)	9 (7.0)	0 (0)	43 (33.6)	
	Moderately	148	18.5	108 (73.0)	36 (24.3)	3 (2.0)	1 (0.7)	40 (27.0)	
	Very	224	28.0	140 (62.5)	69 (30.8)	14 (6.3)	1 (0.4)	84 (37.5)	
	Extremely	156	19.5	98 (62.8)	48 (30.8)	10 (6.4)	0 (0)	58 (37.2)	
Are you concerned about your oral health?	Not at all	227	28.4	184 (81.1)	40 (17.6)	3 (1.3)	0 (0)	43 (18.9)	< 0.001
	Slightly	117	14.6	81 (69.2)	28 (23.9)	8 (6.8)	0 (0)	36 (30.8)	
	Moderately	103	12.9	67 (65.0)	32 (31.1)	4 (3.9)	0 (0)	36 (35.0)	
	Very	216	27.0	149 (69.0)	54 (25.0)	12 (5.6)	1 (0.5)	67 (31.0)	
	Extremely	137	17.1	74 (54.0)	51 (37.2)	11 (8.0)	1 (0.7)	63 (46.0)	
What was your general health status in 2019?	Very Poor	22	2.8	13 (59.1)	7 (31.8)	2 (9.1)	0 (0)	9 (40.9)	< 0.001
	Poor	81	10.1	37 (45.7)	31 (38.3)	12 (14.8)	1 (1.2)	44 (54.3)	
	Acceptable	302	37.8	200 (66.2)	89 (29.5)	13 (4.3)	0 (0)	102 (33.8)	
	Good	293	36.6	222 (75.8)	62 (21.2)	8 (2.7)	1 (0.3)	71 (24.2)	
	Very Good	99	12.4	82 (82.8)	14 (14.1)	3 (3.0)	0 (0)	17 (17.2)	
	Do not know / No answer given	3	0.4	1 (33.3)	2 (66.7)	0 (0)	0 (0)	2 (66.7)	
What was your dental health status in 2019?	Very Poor	9	1.1	5 (55.6)	0 (0)	4 (44.4)	0 (0)	4 (44.4)	< 0.001
	Poor	35	4.4	16 (45.7)	16 (45.7)	3 (8.6)	0 (0)	19 (54.3)	
	Acceptable	346	43.3	225 (65.0)	103 (29.8)	17 (4.9)	1 (0.3)	121 (35.0)	
	Good	331	41.4	246 (74.3)	71 (21.5)	13 (3.9)	1 (0.3)	85 (25.7)	
	Very Good	78	9.8	62 (79.5)	15 (19.2)	1 (1.3)	0 (0)	16 (20.5)	
	Do not know / No answer given	1	0.1	1 (100)	0 (0)	0 (0)	0 (0)	0 (0)	
**Section III: Oral problems in 2019**									
How often did you experience difficulty in eating in 2019?	Never	594	74.3	446 (75.1)	133 (22.4)	13 (2.2)	2 (0.3)	148 (24.9)	< 0.001
	Rarely	87	10.9	48 (55.2)	31 (35.6)	8 (9.2)	0 (0)	39 (44.8)	
	Occasionally	97	12.1	55 (56.7)	32 (33.0)	10 (10.3)	0 (0)	42 (43.3)	
	Frequently	11	1.4	4 (36.4)	4 (36.4)	3 (27.3)	0 (0)	7 (63.6)	
	Very Frequently	9	1.1	1 (11.1)	4 (44.4)	4 (44.4)	0 (0)	8 (88.9)	
	Do not know / No answer given	2	0.3	1 (50)	1 (50)	0 (0)	0 (0)	1 (50)	
Did you experience tooth or gum pain in 2019?	Never	607	75.9	459 (75.6)	132 (21.7)	15 (2.5)	1 (0.2)	148 (24.4)	< 0.001
	Rarely	79	9.9	42 (53.2)	31 (39.2)	6 (7.6)	0 (0)	37 (46.8)	
	Occasionally	92	11.5	47 (51.1)	34 (37.0)	10 (10.9)	1 (1.1)	45 (48.9)	
	Frequently	13	1.6	3 (23.1)	7 (53.8)	3 (23.1)	0 (0)	10 (76.9)	
	Very Frequently	6	0.8	2 (33.3)	0 (0)	4 (66.7)	0 (0)	4 (66.7)	
	Do not know / No answer given	3	0.4	2 (66.7)	1 (33.3)	0 (0)	0 (0)	1 (33.3)	
Did you experience problems with your teeth, mouth or dentition in 2019?	Never	601	75.1	446 (74.2)	132 (22.0)	21 (3.5)	2 (0.3)	155 (25.8)	< 0.001
	Rarely	57	7.1	39 (68.4)	16 (28.1)	2 (3.5)	0 (0)	18 (31.6)	
	Occasionally	101	12.6	52 (51.5)	43 (42.6)	6 (5.9)	0 (0)	49 (48.5)	
	Frequently	14	1.8	5 (35.7)	5 (35.7)	4 (28.6)	0 (0)	9 (64.3)	
	Very Frequently	12	1.5	3 (25.0)	4 (33.3)	5 (41.7)	0 (0)	9 (75.0)	
	Do not know / No answer given	15	1.9	10 (66.7)	5 (33.3)	0 (0)	0 (0)	5 (33.3)	
Did you ever avoid smiling or talking because of the appearance of your teeth or dentition in 2019?	Never	732	91.5	522 (71.3)	181 (24.7)	28 (3.8)	1 (0.1)	210 (28.7)	< 0.001
	Rarely	12	1.5	8 (66.7)	2 (16.7)	2 (16.7)	0 (0)	4 (33.3)	
	Occasionally	37	4.6	20 (54.1)	14 (37.8)	3 (8.1)	0 (0)	17 (45.9)	
	Frequently	12	1.5	5 (41.7)	6 (50)	1 (8.3)	0 (0)	7 (58.3)	
	Very Frequently	7	0.9	0 (0)	2 (28.6)	4 (57.1)	1 (14.3)	7 (100)	
	Do not know / No answer given	0	0	0 (0)	0 (0)	0 (0)	0 (0)	0 (0)	
**Section IV: Tobacco and alcohol consumption**									
Tobacco consumption	I have never smoked	433	54.1	285 (65.8)	123 (28.4)	23 (5.3)	2 (0.5)	148 (34.2)	0.222
	I am a smoker	71	8.9	57 (80.3)	12 (16.9)	2 (2.8)	0 (0)	14 (19.7)	
	I used to smoke	295	36.9	213 (72.2)	69 (23.4)	13 (4.4)	0 (0)	82 (27.8)	
	Do not know / No answer given	1	0.1	0 (0)	1 (100)	0 (0)	0 (0)	1 (100)	
Cigarettes smoked per day	I do not smoke.	729	91.1	498 (68.3)	193 (26.5)	36 (4.9)	2 (0.3)	231 (31.7)	0.605
	I smoke occasionally, not daily.	4	0.5	4 (100)	0 (0)	0 (0)	0 (0)	0 (0)	
	1–5	17	2.1	15 (88.2)	1 (5.9)	1 (5.9)	0 (0)	2 (11.8)	
	6–10	20	2.5	18 (90.0)	2 (10.0)	0 (0)	0 (0)	2 (10.0)	
	> 10	29	3.6	20 (69.0)	8 (27.6)	1 (3.4)	0 (0)	9 (31.0)	
	Do not know / No answer given	1	0.1	0 (0)	1 (100)	0 (0)	0 (0)	1 (100)	
How many years has it been since you gave up smoking?	None	505	63.1	342 (67.7)	136 (26.9)	25 (5.0)	2 (0.4)	163 (32.3)	0.973
	< 5 years	26	3.3	17 (65.4)	7 (26.9)	2 (7.7)	0 (0)	9 (34.6)	
	5–10 years	38	4.8	26 (68.4)	11 (28.9)	1 (2.6)	0 (0)	12 (31.6)	
	11–15 years	29	3.6	19 (65.5)	9 (31.0)	1 (3.4)	0 (0)	10 (34.5)	
	> 15 years	199	24.9	149 (74.9)	41 (20.6)	9 (4.5)	0 (0)	50 (25.1)	
	Do not know / No answer given	3	0.4	2 (66.7)	1 (33.3)	0 (0)	0 (0)	1 (33.3)	
Alcohol consumption	I have never consumed alcoholic beverages	281	35.1	180 (64.1)	83 (29.5)	17 (6.0)	1 (0.4)	101 (35.9)	0.120
	I occasionally consume alcoholic beverages	375	46.9	273 (72.8)	81 (21.6)	20 (5.3)	1 (0.3)	102 (27.2)	
	I consume alcoholic beverages on a daily basis	140	17.5	100 (71.4)	39 (27.9)	1 (0.7)	0 (0)	40 (28.6)	
	Do not know / No answer given	4	0.5	2 (50)	2 (50)	0 (0)	0 (0)	2 (50)	
Beer	0	754	94.3	522 (69.2)	193 (25.6)	37 (4.9)	2 (0.3)	232 (30.8)	0.967
	1	30	3.8	18 (60.0)	11 (36.7)	1 (3.3)	0 (0)	12 (40.0)	
	2	13	1.6	12 (92.3)	1 (7.7)	0 (0)	0 (0)	1 (7.7)	
	3	1	0.1	1 (100)	0 (0)	0 (0)	0 (0)	0 (0)	
	4	1	0.1	1 (100)	0 (0)	0 (0)	0 (0)	0 (0)	
	> 5	1	0.1	1 (100)	0 (0)	0 (0)	0 (0)	0 (0)	
Wine	0	686	85.8	478 (69.7)	169 (24.6)	37 (5.4)	2 (0.3)	208 (30.3)	0.611
	1	62	7.8	35 (56.5)	26 (41.9)	1 (1.6)	0 (0)	27 (43.5)	
	2	30	3.8	23 (76.7)	7 (23.3)	0 (0)	0 (0)	7 (23.3)	
	3	14	1.8	12 (85.7)	2 (14.3)	0 (0)	0 (0)	2 (14.3)	
	4	5	0.6	4 (80.0)	1 (20.0)	0 (0)	0 (0)	1 (20.0)	
	> 5	3	0.4	3 (100)	0 (0)	0 (0)	0 (0)	0 (0)	
Spirits	0	784	98.0	543 (69.3)	201 (25.6)	38 (4.8)	2 (0.3)	241 (30.7)	0.976
	1	12	1.5	8 (66.7)	4 (33.3)	0 (0)	0 (0)	4 (33.3)	
	2	2	0.3	2 (100)	0 (0)	0 (0)	0 (0)	0 (0)	
	3	2	0.3	2 (100)	0 (0)	0 (0)	0 (0)	0 (0)	
**Section V: Dietary habits**									
Do you think a proper diet is important for your oral health?	Not Important	30	3.8	23 (76.7)	6 (20.0)	1 (3.3)	0 (0)	7 (23.3)	0.717
	Slightly Important	24	3.0	18 (75.0)	5 (20.8)	1 (4.2)	0 (0)	6 (25.0)	
	Moderately Important	69	8.6	55 (79.7)	11 (15.9)	3 (4.3)	0 (0)	14 (20.3)	
	Important	318	39.8	224 (70.4)	78 (24.5)	15 (4.7)	1 (0.3)	94 (29.6)	
	Very Important	323	40.4	212 (65.6)	96 (29.7)	14 (4.3)	1 (0.3)	111 (34.4)	
	Do not know / No answer given	36	4.5	23 (63.9)	9 (25.0)	4 (11.1)	0 (0)	13 (36.1)	
How often do you consume white meat?	Never	19	2.4	15 (78.9)	3 (15.8)	1 (5.3)	0 (0)	4 (21.1)	0.500
	Occasionally	114	14.2	86 (75.4)	25 (21.9)	3 (2.6)	0 (0)	28 (24.6)	
	Every four/ five days	226	28.2	149 (65.9)	63 (27.9)	14 (6.2)	0 (0)	77 (34.1)	
	Every two/ three days	413	51.6	284 (68.8)	110 (26.6)	17 (4.1)	2 (0.5)	129 (31.2)	
	Every day	20	2.5	15 (75.0)	2 (10.0)	3 (15.0)	0 (0)	5 (25.0)	
	Do not know / No answer given	8	1.0	6 (75.0)	2 (25.0)	0 (0)	0 (0)	2 (25.0)	
How often do you consume red meat?	Never	84	10.5	58 (69.0)	21 (25.0)	5 (6.0)	0 (0)	26 (31.0)	< 0.001
	Occasionally	264	33.0	176 (66.7)	70 (26.5)	18 (6.8)	0 (0)	88 (33.3)	
	Every four/ five days	261	32.6	178 (68.2)	72 (27.6)	11 (4.2)	0 (0)	83 (31.8)	
	Every two/ three days	175	21.9	131 (74.9)	39 (22.3)	4 (2.3)	1 (0.6)	44 (25.1)	
	Every day	5	0.6	5 (100)	0 (0)	0 (0)	0 (0)	0 (0)	
	Do not know / No answer given	11	1.4	7 (63.6)	3 (27.3)	0 (0)	1 (9.1)	4 (36.4)	
How often do you consume vegetables?	Never	7	0.9	4 (57.1)	3 (42.9)	0 (0)	0 (0)	3 (42.9)	< 0.001
	Occasionally	28	3.5	20 (71.4)	5 (17.9)	3 (10.7)	0 (0)	8 (28.6)	
	Every four/ five days	51	6.4	33 (64.7)	17 (33.3)	1 (2.0)	0 (0)	18 (35.3)	
	Every two/ three days	257	32.1	175 (68.1)	69 (26.8)	13 (5.1)	0 (0)	82 (31.9)	
	Every day	450	56.3	320 (71.1)	108 (24.0)	21 (4.7)	1 (0.2)	130 (28.9)	
	Do not know / No answer given	7	0.9	3 (42.9)	3 (42.9)	0 (0)	1 (14.3)	4 (57.1)	
How often do you consume fruit?	Never	5	0.6	4 (80.0)	1 (20.0)	0 (0)	0 (0)	1 (20.0)	< 0.001
	Occasionally	26	3.3	19 (73.1)	6 (23.1)	1 (3.8)	0 (0)	7 (26.9)	
	Every four/ five days	8	1.0	7 (87.5)	1 (12.5)	0 (0)	0 (0)	1 (12.5)	
	Every two/ three days	43	5.4	31 (72.1)	8 (18.6)	4 (9.3)	0 (0)	12 (27.9)	
	Every day	712	89.0	491 (69.0)	187 (26.3)	33 (4.6)	1 (0.1)	221 (31.0)	
	Do not know / No answer given	6	0.8	3 (50.0)	2 (33.3)	0 (0)	1 (16.7)	3 (50.0)	
How often do you consume legumes?	Never	9	1.1	4 (44.4)	4 (44.4)	1 (11.1)	0 (0)	5 (55.6)	< 0.001
	Occasionally	90	11.3	65 (72.2)	19 (21.1)	6 (6.7)	0 (0)	25 (27.8)	
	Every four/ five days	217	27.1	147 (67.7)	61 (28.1)	9 (4.1)	0 (0)	70 (32.3)	
	Every two/ three days	418	52.3	296 (70.8)	101 (24.2)	20 (4.8)	1 (0.2)	122 (29.2)	
	Every day	57	7.1	38 (66.7)	17 (29.8)	2 (3.5)	0 (0)	19 (33.3)	
	Do not know / No answer given	9	1.1	5 (55.6)	3 (33.3)	0 (0)	1 (50.0)	4 (44.4)	
How much water or liquids do you drink per day?	Less than half a litre	26	3.3	19 (73.1)	6 (23.1)	1 (3.8)	0 (0)	7 (26.9)	< 0.001
	Half a litre	104	13.0	69 (66.3)	29 (27.9)	6 (5.8)	0 (0)	35 (33.7)	
	One litre	262	32.8	184 (70.2)	65 (24.8)	12 (4.6)	1 (0.4)	78 (29.8)	
	One and a half litres	241	30.1	171 (71.0)	62 (25.7)	8 (3.3)	0 (0)	70 (29.0)	
	Two litres	110	13.8	75 (68.2)	28 (25.5)	7 (6.4)	0 (0)	35 (31.8)	
	Over two litres	49	6.1	32 (65.3)	13 (26.5)	4 (8.2)	0 (0)	17 (34.7)	
	Do not know / No answer given	8	1.0	5 (62.5)	2 (25.0)	0 (0)	1 (12.5)	3 (37.5)

### Xerostomia

Some 30.7% of the respondents suffered from xerostomia, most of whom were females (34.8% vs 24% in males) (*p* = 0.003). There were 25.6% with a mild degree of xerostomia, 4.8% moderate and 0.3% severe. The mean XI score was 8.9 ± 6.8. The mean XI was lower with better overall health, so it was 24.6 ± 6.3 (95% CI 19.2–24.8) for poor health, but 17.4 ± 6.3 (95% CI 16.1–18.6) for very good health (*p* < 0.001 Bonferroni test). This pattern was also observed in terms of oral health, with the XI mean recorded as 14.7 ± 10.7 for very poor oral health versus 6.4 ± 5.4 for very good (*p* = 0.002) (Table 2). The full data for the Xerostomia Inventory are found in Table 3.

**Table 2:** XI mean analysis, depending on general and oral health status, daily cigarette consumption and alcohol consumption

	Covariate	N	Mean	SD	CI 95%		P
					Inferior	Superior	
General health	Very Poor	22	22.0	6.29	19.21	24.79	Anova: < 0.001 Bonferroni: P - A: < 0.001 P - G: < 0.001 P - VG: < 0.001 VP - VG: 0.041
	Poor	81	24.64	7.94	22.89	26.40	
	Acceptable	302	20.21	6.56	19.47	20.95	
	Good	293	18.84	6.18	18.13	19.55	
	Very Good	99	17.37	6.31	16.11	18.63	
	Do not know / No answer given	3	24.00	3.60	15.04	32.96	
Oral health	Very Poor	10	14.70	10.68	7.05	22.35	Anova:< 0.001 Bonferroni: VP - VG:0.002 P - VG:0.001 A - VG: 0.001 G - A: 0.008
	Poor	35	11.86	7.14	9.40	14.31	
	Acceptable	346	9.76	6.91	9.03	10.49	
	Good	331	8.04	6.44	7.34	8.74	
	Very Good	78	6.37	5.44	5.14	7.60	
Tobacco consumption	I have never smoked	433	9.50	7.02	8.84	10.16	Anova: 0.006 Bonferroni: N - S: 0.020
	I am a smoker	71	7.14	5.72	5.79	8.50	
	I used to smoke	295	8.33	6.58	7.58	9.09	
Alcohol consumption	I have never consumed alcoholic beverages	281	20.63	7.11	19.79	21.47	Anova:0.063 Bonferroni: N - D: 0.050
	I occasionally consume alcoholic beverages	375	19.68	6.85	18.99	20.38	
	I consume alcoholic beverages on a daily basis	140	18.81	5.79	17.85	19.78	
	Do not know / No answer given	4	21.00	6.73	10.29	31.71	

Table legend: Anova and Bonferroni test: Very Poor (VP), Poor (P), Acceptable (A), Good (G), Very Good (VG), I have never smoked (N), I am a smoker (S), I used to smoke (E), I have never consumed alcoholic beverages (N), I occasionally consume alcoholic beverages (O), I consume alcoholic beverages on a daily basis (D)

**Table 3:** Full XI data. Responses to individual items

	N (%)					
	Never	Hardly ever	Occasionally	Fairly often	Very often	Missing
My mouth feels dry	337 (42.1)	107 (13.4)	218 (27.3)	80 (10)	57 (7.1)	1 (0.1)
I have difficulty eating dry food	578 (72.3)	73 (9.1)	82 (10.3)	32 (4.0)	25 (3.1)	10 (1.3)
I wake up at night to drink water or other liquids.	499 (62.4)	66 (8.3)	147 (18.4)	39 (4.9)	47 (5.9)	2 (0.3)
My mouth feels dry when I am chewing food.	650 (81.3)	86 (10.8)	43 (5.4)	10 (1.3)	8 (1.0)	3 (0.4)
I need to drink liquids when I am swallowing food.	472 (59.0)	77 (9.6)	149 (18.6)	56 (7.0)	40 (5.0)	6 (0.8)
I have difficulty swallowing certain foods.	699 (87.4)	34 (4.3)	56 (7.0)	6 (0.8)	4 (0.5)	1 (0.1)
The skin on my face is dry.	428 (53.5)	96 (12.0)	130 (16.3)	63 (7.9)	70 (8.8)	13 (1.6)
I need to suck sweets or similar to relieve the dry mouth sensation.	609 (76.1)	47 (5.9)	99 (12.4)	28 (3.5)	15 (1.9)	2 (0.3)
My eyes are dry.	427 (53.4)	77 (9.6)	174 (21.8)	75 (9.4)	42 (5.3)	5 (0.6)
My lips are dry.	418 (52.3)	114 (14.2)	181 (22.6)	47 (5.9)	37 (4.6)	3 (0.4)
The inside of my nose feels dry.	427 (53.4)	77 (9.6)	174 (21.8)	75 (9.4)	42 (5.3)	5 (0.6)

### General and oral health

There were 74.4% of respondents reporting acceptable or good general health and only 12.9% stated that their health was poor or very poor. The incidence of xerostomia in people with poor general health was 54.3% as opposed to 17.2% in people with good health (*p* < 0.001). There were 84.7% of respondents reporting acceptable or good oral health, with only 5.5% reporting that their oral health was poor or very poor. Of those who claimed to have poor oral health, 54.3% had xerostomia, unlike 20.5% of those who considered that their oral health is very good (*p* < 0.001). Xerostomia degree was higher among those who expressed greater concerns about their oral health, with an incidence of 46% among those who expressed great concern about this aspect, compared with just 18.9% of those who stated that they were not concerned about their oral health (*p* < 0.001). With regards to quality of life, 15.9% reported that they had problems with their mouth, teeth or dentition; 14.6% that they had difficulty eating; 13.9% that they experience tooth or gum pain, and 7% that they had avoided smiling or talking because of the appearance of their teeth or dentition. The percentage of xerostomia was higher (*p* < 0.001) the higher the frequency with which oral issues were suffered. In fact, 100% of people who stated that they avoided smiling or talking on a very frequent basis had a certain degree of xerostomia, whereas 28.7% stated that they never avoided said actions (p < 0.001).

### Health risk behaviours: tobacco, alcohol and dietary habits

There were 54.1% who stated that they had never smoked, while 36.9% declared that they were ex-smokers, with the majority having given up smoking more than 15 years ago, and only 8.9% stated that they currently smoke. The number of non-smokers was higher among women and among those over the age of 74 years (*p* < 0.001).

Of those interviewed, 46.9% stated that they consume alcoholic beverages occasionally, and 17.5% that they do so on a daily basis, mainly wine, consuming around 1 or 2 drinks a day (7.8 and 3.8% respectively). Furthermore, 35.1% stated that they do not consume any alcohol at all, and this percentage was higher among women (47.5% vs 18.2%) (p < 0.001). With regards to tobacco and alcohol consumption, no significant differences were observed in terms of xerostomia.

Most of the participants stated that they consume fruit and vegetables on a daily basis (89 and 56.3% respectively), as opposed to their less frequent consumption of meat and legumes (only 0.6% stated that they consume red meat every day and, likewise, this figure was 2.5% for white meat, and 7.1% for legumes). As regards the consumption of red meat, there was a significant difference between men and women (*p* < 0.001), with the latter consuming less red meat. Among those who stated that they never consume vegetables, the percentage of xerostomia was 42.9%, but 28.9% for those who consume said products on a daily basis (*p* < 0.001). The incidence of xerostomia among individuals who stated that they never consume legumes was 55.6% but 29.2% for those who consume legumes every two or three days (p < 0.001).

**Fig. 2:**
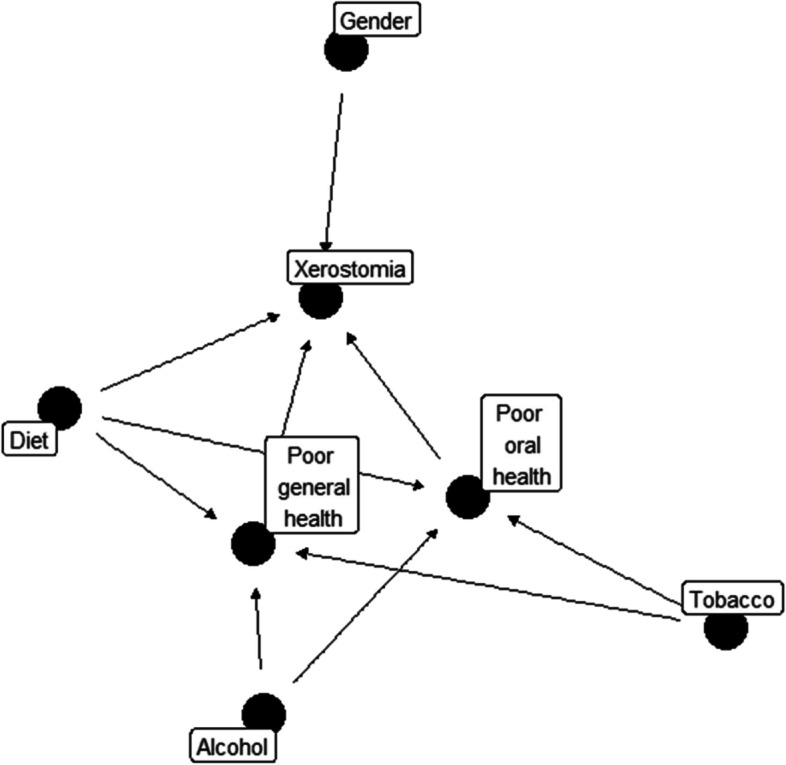
Directed Acyclic Graph (DAG)

The relation between the main studied variables has been illustrated in Fig. 2 by a DAG. The influence of general and oral health and health risk behaviours can be graphically observed.

### Logistic regression analysis

Logistic regression showed that the highest OR for xerostomia was observed among adults with poor general health, who had 2.81 higher odds of suffering from this condition (95%CI 1.8–4.3, *p* < 0.001) than those in good health. In the model adjusted for gender, oral health, education, employment and household chores, the OR was 2.18 (95%CI 1.4–3.4, *p* = 0.001). Those who need help with household chores had 2.16 higher odds of suffering xerostomia (95%CI 1.4–3.4; p = 0.001) and the OR was 1.69 (95%CI 1.1–2.7; *p* = 0.03) in the adjusted model. Females had higher odds of suffering from xerostomia than males in both the univariate and multivariate models. Overall, adults with poor oral health had higher odds of suffering from this condition (Table 4).

**Table 4:** A univariate logistic regression analysis was performed to determine the univariate OR *(Odds Ratio)* for xerostomia. The adjusted statistical analysis was performed using multivariate logistic regression corrected for gender, general health and oral health, education, employment, and household chores

	Univariate (OR 95% CI)	***p*** value	Adjusted (OR 95% CI)	p value
**Gender**				
Female vs male	1.68 (1.22–2.32)	0.001	1.41 (0.99–1.99)	0.05
**General health**				
Poor vs good	2.81 (1.84–4.28)	< 0.001	2.18 (1.38–3.44)	0.001
**Oral health**				
Poor vs good	2.63 (1.42–4.84)	0.002	1.79 (0.92–3.49)	0.86
**Education**				
Uneducated vs Primary Education	1.31 (0.65–2.63)	0.44	1.48 (0.71–3.05)	0.28
Uneducated vs Higher Education	0.77 (0.38–1.58)	0.48	1.04 (0.49–2.22)	0.91
Uneducated vs First Level Vocational Education	1.31 (0.59–2.91)	0.50	1.67 (0.73–3.84)	0.22
Uneducated vs Higher-level Vocational Education and Training	0.73 (0.34–1.55)	0.41	0.90 (0.41–1.98)	0.81
**Employment**				
Self-employed vs employed	1.88 (0.94–3.79)	0.76	1.52 (0.73–3.16)	0.26
Self-employed vs others	1.11 (0.74–1.68)	0.61	1.15 (0.75–1.76)	0.51
**Household chores**				
Require help vs self-sufficient	2.16 (1.39–3.37)	0.001	1.69 (1.05–2.74)	0.03

## Discussion

Our findings indicate a strong association between xerostomia (assessed using XI) and general and oral health. We found that the prevalence of xerostomia was 30.6%. Women had a higher risk of suffering from xerostomia than men. Poor general and oral health have been reported as risk factors for xerostomia. Regarding alcohol and tobacco consumption, the results were quite heterogeneous and we did not observe any variation in relation to xerostomia. A lower perception of xerostomia was observed among those who consume vegetables and legumes on a regular basis.

When interpreting the findings of this study it is important to consider certain limitations. Firstly, given its cross-sectional nature, it is not possible to prove causality, which would be ideal for clinical translation, therefore emphasizing the need for prospective longitudinal studies. Nevertheless, this research has notable strengths, such as a large sample size and the fact that our findings were drawn from a very specific group by age range (over 65 years of age) residing in different areas of Spain.

The prevalence of xerostomia was close to the findings reported by other international organizations, which put it between 20 and 30% [18]. In Australia, an incidence of 26.5% was reported among people over 75 years of age [19]. Among participants aged 20 to 80 years, Nederfors et al., observed a significant difference in incidence (21.3% in men and 27.3% in women) which increased substantially with age [18]. A recent prospective study among younger participants (aged 20–59 years) revealed that general health affects episodes of xerostomia [20]. Although in our study the majority of the respondents claimed to have an acceptable or good oral health status, in the systematic review by Wong et al. [21], the oral hygiene and oral health of older adults was reported to be poor. The explanation for this apparent contradiction could be that self-perceived health is often better than objectively observed health. Furthermore, self-reporting of general and oral health, as well as health risk behaviours may be biased by social desirability, which may lead to inaccurate self-reports and erroneous conclusions. Heberto et al. [22] have observed a high bias in reporting food intake. However, in a study published in 2020 [23], it has been reported that there is no significant association between social desirability bias and general medical beliefs or self-reported health. Methods to decrease this bias include writing and prefacing questions [24], which were designed by gerodontology experts in this study. In terms of the quality of life, as other authors have also observed [25, 26], xerostomia has a significant negative impact on the older population’s quality of life.

According to the “Global Status Report on Alcohol and Health” 2018 [27], 43% of the world’s population are current drinkers, while in our sample 64.4% reported drinking alcohol occasionally or daily. Wine was the most consumed beverage (14.4% of daily consumers), followed by beer, while only 2.1% stated that they consume spirits every day. This consumption pattern is very different to the one observed worldwide, where 44.8% of all recorded alcohol consumption was in the form of spirits, followed by beer and wine [27]. Several studies have shown that the use of alcohol or alcohol-free mouthwashes does not significantly affect xerostomia [28, 29]. We also did not find any relevant differences in terms of tobacco use and xerostomia severity, although preliminary studies have found that smoking significantly increases symptoms of xerostomia [8]. In studies of younger populations (under 60 years of age), smoking has also been found to increase the likelihood of suffering from regular xerostomia [20]. Xerostomia’s relationship with tobacco and alcohol remains unclear [30], however, whether or not these health risk behaviours play a relevant role in the development of xerostomia, there should be avoided as there  is evidence that they do play a significant role in the development of oral cancer and other systemic diseases. A healthy diet should be advised and collaboration from doctors and dentists is essential in dietary interventions; indeed, dentists may be the earliest healthcare providers to detect an eating disorder [31]. Dentistry enables older adults to follow a satisfactory diet by restoring dental function and, as oral health improves, there is an opportunity to promote a good diet among this population group.

A cooperative approach involving different healthcare professionals in geriatric caregiving makes it possible to adjust to the individual needs of older patients [32, 33]. In this sense, our study updated and built on the current knowledge on the subject by providing evidence of xerostomia’s relationship with general and oral health in Spain. Although, mild cognitive impairment can be assumed in the participants, it was not assessed objectively as it requires a clinical diagnosis aided by a complete medical record, neurological examination, mental status examination and formal neuropsychological testing [34].

Our findings can probably be extrapolated to the rest of Europe. In Norway and Sweden, a dramatically increased incidence of xerostomia has also been reported amongst older patients, which must be taken into account in the clinical management of these individuals. It has also been pointed out that the comorbidity between xerostomia and oral pathologies must not be ignored in older adults [35–37].

In conclusion, we found a strong association between general and oral health with xerostomia in older adults, so this relationship should be taken into account when providing health care to this group. The findings of our study showed the value of focusing on general and oral health when detecting xerostomia in older people, as well as periodic assessment of xerostomia in patients with poor health.

## Supplementary Information

**Additional file 1.** Survey database.

## Data Availability

The dataset supporting the conclusions of this article is included within the article (and its additional file).
